# A Cross-Protective Vaccine Against 4b and 1/2b *Listeria monocytogenes*

**DOI:** 10.3389/fmicb.2020.569544

**Published:** 2020-12-11

**Authors:** Fanzeng Meng, Tengfei Zhu, Hao Yao, Zhiting Ling, Youwei Feng, Guo Li, Jing Li, Xinyu Sun, Jiaqi Chen, Chuang Meng, Xin’an Jiao, Yuelan Yin

**Affiliations:** ^1^Jangsu Key Laboratory of Zoonosis, Yangzhou University, Yangzhou, China; ^2^Key Laboratory of Prevention and Control of Biological Hazard Factors (Animal Origin) for Agrifood Safety and Quality, The Ministry of Agriculture of China, Yangzhou University, Yangzhou, China; ^3^Joint International Research Laboratory of Agriculture and Agri-Product Safety of the Ministry of Education of China, Yangzhou University, Yangzhou, China; ^4^Jiangsu Co-innovation Center for the Prevention and Control of Important Animal Infectious Disease and Zoonosis, Yangzhou University, Yangzhou, China

**Keywords:** *Listeria monocytogenes*, attenuated vaccine, cellular immune response, cross protection, humoral immune response

## Abstract

*Listeria monocytogenes* (*Lm*) is a foodborne zoonotic pathogen that causes listeriosis with a mortality rate of 20–30%. Serovar 4b and 1/2b isolates account for most of listeriosis outbreaks, however, no listeriosis vaccine is available for either prophylactic or therapeutic use. Here, we developed a triple-virulence-genes deletion vaccine strain, and evaluated its safety, immunogenicity, and cross-protective efficiency. The virulence of NTSNΔ*actA*/*plcB*/*orfX* was reduced 794-folds compared with the parental strain. Additionally, it was completely eliminated in mice at day 7 post infection and no obvious pathological changes were observed in the organs of mice after prime-boost immunization for 23 days. These results proved that the safety of the *Lm* vaccine strain remarkably increased. More importantly, the NTSNΔ*actA*/*plcB*/*orfX* strain stimulated higher anti-Listeriolysin O (LLO) antibodies, induced significantly higher expression of IFN-γ, TNF-α, IL-17, and IL-6 than the control group, and afforded 100% protection against serovar 4b and 1/2b challenges. Taken together, our research demonstrates that the triple-genes-deletion vaccine has high safety, can elicit strong Th1 type immune response, and affords efficient cross-protection against two serovar *Lm* strains. It is a promising vaccine for prevention of listeriosis.

## Introduction

*Listeria monocytogenes (Lm)* was first isolated by Murray, Webb, and Swann from dead laboratory rabbits and guinea pigs in 1926 (Murray et al., 2005). It is a gram-positive, foodborne bacterium that causes severe listeriosis in immunosuppressed populations, including the elderly, pregnant women, and newborns (Buchanan et al., 2017). Listeriosis is associated with miscarriage, gastroenteritis, sepsis, and meningitis, with a mortality rate of 20–30% (Hernandez-Milian and Payeras-Cifre, 2014; Schutte et al., 2019). Listeriosis has remained a problem in recent years, listeriosis outbreaks were reported in South Africa (Smith et al., 2019), the United States (Angelo et al., 2017), the European Union (Calderon-Gonzalez et al., 2017; Duranti et al., 2018), and other countries and regions. The emergence of hypervirulent strains, as well as extensively drug-resistant and multi-drug resistant clinical isolates, has brought new challenges to prophylactic and therapeutic treatment of listeriosis (Camargo et al., 2015; Jahan and Holley, 2016). The field has advanced our understanding of *Lm* physiology and infection process considerably (Mostowy and Cossart, 2012; Rolhion and Cossart, 2017).

*Lm* is a facultative intracellular pathogen that can escape from phagocytic vesicles into the host cytoplasm, where it activates NF-κb pathways, and modulates the type I interferon response. Thus, *Lm* regulates the host’s innate and adaptive immune responses and decreases host resistance to systemic infection (Stockinger et al., 2004; Opitz et al., 2006; Boneca et al., 2007). In the process of host-*Lm* interactions, *Lm* virulence factor listeriolysin O (LLO) can dissolve phagocytic vesicles to assistant bacteria escaping into the cytoplasm of phagocytes (Nguyen and Portnoy, 2020). *Lm* antigens were efficiently presented to the MHC-I and MHC-II processing pathways, thus presenting antigenic peptides to either CD4^+^ T cells or CD8^+^ T cells (D’Orazio, 2019). The activated T cells differentiate and generate the adaptive immune response, inducing the production of the Th1 cytokines IFN-γ, TNF-α, and IL-12 (Nomura et al., 2002; Berg et al., 2005). The activated CD8^+^ cytotoxic lymphocytes secrete perforin, granzyme, and granlysin and so on, thus killing the infected cells along with intracellular *Lm* (Ito et al., 2015; Lin and Flynn, 2015). IL-17 is a pro-inflammatory factor, involved in regulating immune response to promote multiple chemokines and inflammatory cytokines, it is essential in defense of the host against the pathogen (Freches et al., 2013; Chamoun et al., 2018). IL-10 is an immunosuppressive and anti-inflammatory substance, plays a vital role in enhancing secretion of anti-inflammatory modulators and specifically inhibiting the cellular immune response (Murai et al., 2009). The variant production between pro-inflammatory cytokines and anti-inflammatory cytokines reflects the biases of immune response against pathogen. *Lm* mediates efficient cellular immune responses and memory T cell responses, thus consistently inducing robust protective immunity. Therefore, *Lm* as a vaccine vector of delivering foreign antigens has been intensively studied, in particular for tumor therapy vectors (McLaughlin et al., 2013; Crittenden et al., 2015; Ding et al., 2019). Although the *Lm* vectored vaccine have inspiring therapeutic effect in clinic, until now, no vaccine is available to protect high-risk populations against listeriosis.

Vaccines is an effective way to prevent and treat listeriosis. Among available *Listeria* vaccines, live attenuated vaccines are highly preferred, since they are highly immunogenic, stimulate robust cellular immunity, and provide longer immune-protection. Knocking out *Lm* virulence and virulence-associated genes is the primary strategy to reduce its virulence. Several virulence genes are required for a successful *Lm* systemic infection, which are harbored in the *Listeria* pathogenic island 1 (LIPI-1). These genes enable *Lm* to invade cells (*prfA*), escape from phagocytic vesicles (*hly*, *mpl*, *plcA*, *plcB*), survive in the cytoplasm (*orfX*), and facilitate polar movement and cell-to-cell spread (*actA*) (de las Heras et al., 2011). Studies have shown that knocking out LIPI-1 genes can significantly reduce *Lm* virulence (Yin et al., 2008; Vasanthakrishnan et al., 2015). For example, the *Lm*Δ*actA* strain is unable to spread from cell to cell, significantly reducing its virulence compared to the wild-type strain (Saklani-Jusforgues et al., 2003). Our previous research reported the development of a serovar 1/2a *Lm*Δ*actA*/*plcB* strain, which provided effective protection against the wild-type strain challenge (Yin et al., 2008). A triple mutant strain *Lm*ddA with deletion *actA*, *dal*, and *dat* generated protective immunity against a lethal challenge (Thompson et al., 1998). Encouragingly, the researchers developed the Δ*inlB*Δ*actA* vaccine for which Phase I safety trials indicated no adverse effects in humans (Johnson et al., 2011). However, the bottleneck for the development of an attenuated *Listeria* vaccine is maintaining the balance between safety and virulence. Variations of the ideal attenuated vaccines, those with high safety and robust protective immunity, are on the way.

Based on somatic and flagellar antigens, fourteen serotypes have been identified for *Lm*, comprising 1/2a, 1/2b, 1/2c, 3a, 3b, 3c, 4a, 4ab, 4b, 4c, 4d, 4e, 4h, and 7 (Jamshidi and Zeinali, 2019; Yin et al., 2019). As we know, more than 80% of listeriosis in animals and about 50% of listeriosis in humans are caused by serotype 4b *Lm*, which usually manifests as severe miscarriage or neurological symptoms (Czuprynski et al., 2002; Miya et al., 2008; Yao et al., 2018). Serotype 4b strain is primary cause of human and animal listeriosis (Kathariou et al., 2006; Walecka-Zacharska et al., 2013). In this study, we used the serotype 4b strain *Lm* NTSN, which was isolated from a sheep listeriosis outbreak, and constructed a mutant strain with the deletion of three neighboring genes in LIPI-1, *actA*, *plcB*, and *orfX*. Using this triple-genes-deletion mutant strain, we characterized the cellular and humoral immunity as well as protective immunity induced in a murine model of *Lm* systemic infection.

## Materials and Methods

### Bacterial Strains and Materials

The NTSN (4b serotype *Lm*) and DH5α (*Escherichia coli*) strains were obtained from the Jiangsu Key Laboratory of Zoonosis. The Yc32 (1/2b serotype *Lm*) was isolated from mutton in Yangzhou, Jiangsu, and was stored in the Jiangsu Key Laboratory of Zoonosis. The pAULA vector was a gift from professor Trinad Chakraborty (University of Giessen, Germany). Brain heart infusion (BHI) broth was purchased from Becton-Dickinson Company (BD, United States).

### Animals and Ethics Statement

6 weeks old female BALB/c and C57BL/6 mice were purchased from Vital River Laboratory Animal Technology Co., Ltd. (Beijing, China). All animal experiments were performed according to the Laboratory Animal-Guideline for Using Animals in the Education of the Chinese Association for Laboratory Animal Sciences (CALAS). The animal study was reviewed and approved by Yangzhou University Institutional Animal Ethics Committee (Yangzhou, China).

### Construction of the Triple-Genes-Deletion Mutant

To achieve the *actA*, *plcB*, and *orfX* deletion strain, the recombinant plasmid pAULA-*actA*-U/*orfX*-D was constructed with the primers listed in Table 1. The upstream homology fragment, *actA*-U, consisted of the 447 bp sequence of *mpl* and the insertion sequence (IS), located between *actA* and the 198 bp *mpl*. The downstream homology fragment, *orfX*-D, consisted of NTSN_0217 (333 bp) and a 179 bp IS. The ClonExpress^TM^ II One Step Cloning Kit (Vazyme, Nanjing, China)was used to ligate *actA*-U and *orfX*-D fragments with linearized pAULA plasmid. Following identification of the desired clone, the recombinant pAULA plasmid was electroporated into NTSN competent cells. The mutant strain NTSN Δ*actA/plcB/orfX* was obtained according to previously described methods (Yin et al., 2008).

**TABLE 1:** Primers used in this study.

**Name**	**Sequence (5′–3′)^a^**	**Application**
*acA* +	ACGACGTTGTAAAACGACGGCCAGTTTAGAATACGAAGGGCAATCAG	Mutant constructed
*actA* −	CGCTCGTGTTCATTCAAAATTCTTATACTCCCTCCTCGTGAT	Mutant constructed
*orfX* +	ATCACGAGGAGGGAGTATAAGAATTTTGAATGAACACGAGCG	Mutant constructed
*orfX*−	TTACGCCAAGCTTGCATGCCTGCAGATCACCGTTTGAAGACATACCAGGG	Mutant constructed
SN-F	GTCAGCGGATGAGTCTACACCACAA	Mutant verification
SN-R	TTTGGATTACTGGTAGGCTCGGCAT	Mutant verification
SW-F	GTGTCCTTAACTCTCTCTGTCA	Mutant verification
SW-R	ACAAGCCTTAGAAAACCCCAAT	Mutant verification
*actA*-F	AGCGGATGAGTCTACACCACAA	RT-PCR
*actA*-R	GATTACTGGTAGGCTCGGCATA	RT-PCR
*plcB*-F	AGCAAGTGCCTGTTGTGATG	RT-PCR
*plcB*-R	ACAGTGGTAGCCTGGTGGAT	RT-PCR
*orfX*-F	ATTCTTATCCACTCGTTAGCGG	RT-PCR
*orfX*-R	GTTCATTCAAAATTCCAGCCAT	RT-PCR
*gyrB*-F	TAATGCTCTTTCCACATCTCTTG	RT-PCR
*gyrB*-R	CGGTAATCCGTTTCGCCT	RT-PCR
IFN-γ-F	GGAACTGGCAAAAGGATGGT	qRT-PCR
IFN-γ-R	ACGCTTATGTTGTTGCTGATGG	qRT-PCR
L-17-F	AAACACTGAGGCCAAGGAC	qRT-PCR
L-17-R	CGTGGAACGGTTGAGGTAG	qRT-PCR
TNF-α-F	TCTCATTCCTGCTTGTGG	qRT-PCR
TNF-α-R	ACTTGGTGGTTTGCTACGA	qRT-PCR
IL-10-F	ACCTGGTAGAAGTGATGCC	qRT-PCR
IL-10-R	GACACCTTGGTCTTGGAG	qRT-PCR
IL-4-F	TCACAGCAACGAAGAACACC	qRT-PCR
IL-4-R	CGAAAAGCCCGAAAGAGTC	qRT-PCR
IL-6-F	TACCACTCCCAACAGACC	qRT-PCR
IL-6-R	CATTTCCACGATTTCCCAGA	qRT-PCR
GAPDH-F	CAAATTCAACGGCACAGTCA	qRT-PCR
GAPDH-R	TTAGTGGGGTCTCGCTCC	qRT-PCR

*^a^The actA + and orfX- primers contain homologous fragments of the pAULA vector which sequences marked by an underscore.*

### Confirmation of the Triple-Genes-Deletion Mutant

#### RT-PCR

NTSNΔ*actA/plcB/orfX* and wild-type NTSN were overnight cultured at 37°C, then collected by centrifugation. RNA was extracted using the RNeasy Plus Mini Kit (Qiagen, Germany). The cDNA of triple-genes-deletion and wild-type strain was obtained according the PrimeScript RT reagent kit protocol (Takara, China). The primers listed in Table 1 were used to amplify fragments of *actA*, *plcB, orfX*, and *gyrB.*

#### Western Blotting

The cell wall proteins of NTSNΔ*actA/plcB/orfX* and NTSN were extracted and analyzed by SDS-PAGE, followed by Western blot. Monoclonal antibodies against either LLO or ActA were used as the primary antibody, while HRP-(horseradish peroxidase)-labeled goat anti-mouse IgG was used as the secondary antibody, and diaminobenzidine (DAB) was the HRP substrate for Western blot analysis.

#### Phospholipase C Activity

Based on a previous report, we made egg yolk agar charcoal plates to verify phospholipase C activity of our strains. Here, 0.5 g activated charcoal was added to 100 mL of BHI agar, the pH adjusted to 6.5, and then autoclaved (Yeung et al., 2005). Afterward, 8 mL of bacteria-free chicken egg yolk was added to the molten medium (45°C) and the mixture was poured into Petri dishes. Wild-type (WT) and triple-genes-deletion strains were streaked on the plate surface and incubated at 37°C for 48 h.

### Genetic Stability of the Triple-Genes-Deletion Mutant

A single colony of NTSNΔ*actA/plcB/orfX* was inoculated in BHI liquid and cultured at 37°C for 20–24 h as the first passage. This step was repeated until the 30th generation had been passaged.

#### Detection of Deleted Genes

The bacterial genome extraction kit was used to extract the genomes of each strain subline. Those genomes were prepared as the template for PCR amplification with outer primers (SW-F/R) and inside primers (SN-F/R) (Table 1). Wild-type NTSN was used as a positive control to demonstrate if deleted genes were restored in either the original NTSNΔ*actA/plcB/orfX* strain, or in strain sublines at 5, 15, and 30 generation passages.

#### Genetic and Biochemical Identification of the Serially Passaged Strains

We inoculated the original NTSNΔ*actA/plcB/orfX* strain and strain sublines at 5, 15, and 30 generation passages on BHI plate and incubated them at 37°C for 12–16 h. A single colony was inoculated in PBS to obtain a bacterial suspension. The optical density (OD) of the bacterial suspension was adjusted to McFarland 0.50–0.63 using a turbidimetric instrument. The 1.8 mL suspension of each strain was added to a VITEK 2 gram-positive biochemical identification card and put into the automatic biochemical identification instrument (bioMérieux, France) to analyze 43 biochemical indicators of *Lm*.

### Evaluation the Virulence of NTSNΔ*actA/plcB/orfX*

#### Determination of LD_50_

Six weeks old BALB/c female mice were randomly divided into six groups (5 mice/group) and infected by NTSNΔ*actA/plcB/orfX* and NTSN. The NTSN Δ*actA/plcB/orfX* and NTSN were inoculated in BHI broth and cultured for 12 h at 37°C. The culture was diluted 1:40 with fresh broth and then transferred to 10 mL of fresh BHI liquid medium and cultured until it reached an OD_600_ value of 0.8. The LD_50_ of NTSN was determined by intraperitoneal injection. The remaining bacteria were plated on BHI agar for bacterial colony counts. The mice were monitored for next 14 days to determine the median lethal dose (LD_50_).

#### Assessment of Bacterial Loads

Six weeks old BALB/c female mice were randomly divided into three groups (30 mice/group) and infected abdominally with either NTSNΔ*actA/plcB/orfX* (5 × 10^5^ CFU), NTSN wild-type (5 × 10^3^ CFU), or PBS (150 μL). On day 1, 3, 5, 7, and 9 post-infection, the spleens and livers were removed from each group, homogenized, and cultured for bacterial colony counts.

Six weeks old C57BL/6 and BALB/c female mice were randomly divided into four groups (6 mice/group) and infected abdominally with NTSNΔ*actA/plcB/orfX* (5 × 10^5^ CFU) or NTSN wild-type (5 × 10^3^ CFU). On day 3 post infection, the spleens and livers were harvested and homogenized. Next, the samples were cultured for bacterial colony counts. Mice were weighed before infection and after infection for 3 days post-infection to compare the changes in body weight.

#### Histopathological Analysis

Six weeks old BALB/c female mice were randomly separated into two groups (6 mice/group) and immunized subcutaneously with either NTSNΔ*actA/plcB/orfX* (5 × 10^5^ CFU) or PBS (150 μL) followed, by a booster on day 14 post-vaccination (Table 2). The mice were sacrificed 14 days after the booster immunization. The spleen, liver, kidney, heart, lungs, and brain of each mouse were prepared for histopathological sections, and subjected to HE staining at WuXi AppTec Co., Ltd.

**TABLE 2:** Mouse immunization and challenge procedures via intraperitoneal (i.p) injection.

**Groups**	**Number of immunization bacteria (CFU/mouse)**	**Dose of immunization (μL/mouse)**	**Number of challenge bacteria (CFU/mouse)**	**Dose of challenge (μL/mouse)**
A	PBS/0	150	2.5 × 10^6^	150
B	PBS/0	150	7 × 10^5^	150
C	5 × 10^6^	150	2.5 × 10^6^	150
D	5 × 10^6^	150	7 × 10^5^	150
E	PBS/0	150	–	–
F	5 × 10^6^	150	–	–
G	5 × 10^3^	150	–	–

*A, The PBS inoculated group challenged by Yc32; B, The PBS inoculated group which challenged by NTSN wild-type strain; C, The NTSNΔactA/plcB/orfX immunized group challenged by Yc32 strain; D, The NTSNΔactA/plcB/orfX immunized group challenged by NTSN wild-type strain; E, C57BL/6 mice were inoculated by PBS for cellular immune response determination; F, C57BL/6 mice were immunized by NTSNΔactA/plcB/orfX for cellular immune response determination; G, C57BL/6 mice were inoculated by NTSN for cellular immune response determination.*

### Detection of Antibodies in the Serum of Immunized Mice

Six weeks old BALB/c female mice in each group (6 mice/group) were immunized subcutaneously with NTSN, NTSNΔ*actA/plcB/orfX* or PBS, and given a boost with the same dose 14 days subsequent the first immunization (Table 2). The humoral immunse response induced by the inoculation of NTSNΔ*actA/plcB/orfX* in mice was evaluated by indirect enzyme-linked immunosorbent assay (ELISA). Blood samples were collected at day 7, 14, 21, and 28. LLO was diluted in NaHCO_3_ buffer (10 mmol/L, pH 9.6) and antibody-coated at a final concentration of 0.64 μg/mL. HRP-labeled goat anti-mouse IgG1 and IgG2a (1:5,000; Sigma, United States) were used as secondary antibody. The absorbance rate at OD_45__0n__*m*_ was detected by multifunctional microplate detector (BioTek, Berten, United States) and a P (Sample OD_450_)/N (negative control OD_450_) greater than 2.1 was considered positive.

### Cellular Immune Response of Prime-Boost Immunization

Six weeks old C57BL/6 mice were randomly divided into three groups (5 mice/group) and subcutaneously immunized with 0.1 LD_50_ NTSN*ΔactA/plcB/orfX*, wild-type NTSN or PBS (Table 2). On day 7 after the second immunization, spleen of C57BL/6 mice were harvested and extracted total RNA using RNAprep Pure Tissue Kit (Tiangen, China), then transcripted to cDNA using PrimeScript RT reagent Kit (Takara, China). Next, the transcriptional expression of IL-4, IL-6, IL-10, IFN-γ, TNF-α, and IL-17A cytokines were determined by quantitative Real-Time PCR (qRT-PCR). The primers of cytokines used by qRT-PCR are shown in Table 1. The cDNA template was diluted 4 times with RNA free ddH_2_O. Cycling conditions were 95°C for 30 s, followed by 40 cycles at 95°C for 5 s, and 60°C for 30 s. The Real-time PCR reactions were performed on 7500 Real Time PCR System (Applied Biosystems qPCR; Thermo Fisher Scientific, United States). Expression levels [relative quantification (RQ)] were assessed using 2^–ΔΔ*CT*^ method.

### Challenge With Hypervirulent *Lm* Strains

The immunization efficacy of NTSN*ΔactA/plcB/orfX* was further evaluated by a challenge experiment. Six weeks old female C57BL/6 mice were randomly divided into four groups (6 mice/group), where two groups were intraperitoneally inoculated with PBS, and other two with the mutant strain (Table 2). All groups were prime-boost immunized in 14 days intervals. On day 14 after the second immunization, they were challenged with 10 LD_50_ *Lm* NTSN or *Lm* Yc32 (Table 2). Mice were monitored daily for 2 weeks post-challenge.

### Software of Statistical Analysis

Statistical analyses and statistical drawing were performed with Prism 8 (GraphPad Software, version 8). One-way ANOVA pairwise compares the means from two or more groups in pairs to the relative means of control group; two-way ANOVA with Tukey’s or Sidak’s multiple comparisons test was used to compare the means of two groups. Statistically significant differences are: ^∗^*P* < 0.05; ^∗∗^*P* < 0.01; ^∗∗∗^*P* < 0.001; ^****^P < 0.0001. Statistically non-significant (ns) was denoted when *p*-value was > 0.05. The primers are designed by Primer Premier 5.0, which produced Premier Biosoft International, Canada.

## Results

### The Mutant Strain Was Successfully Constructed

**FIGURE 1:**
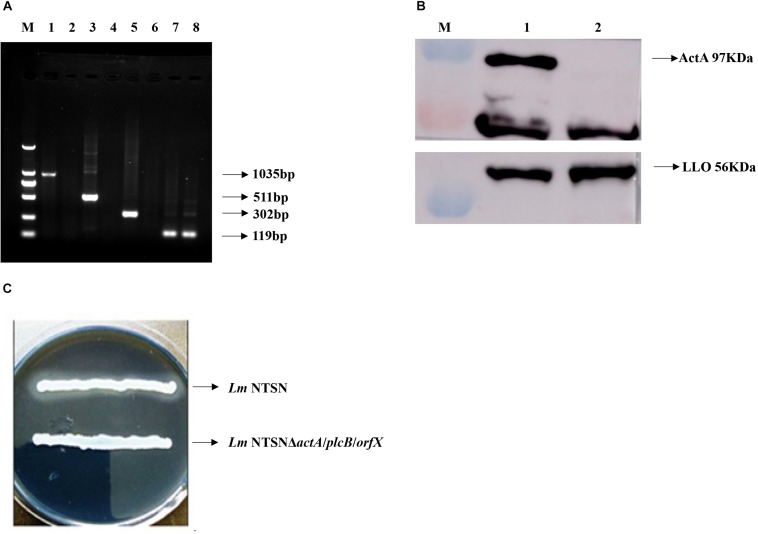
Verification of each virulence gene deletion. **(A)** RT-PCR analysis to verify the deletion of *actA*, *plcB*, and *orfX*. M represented DNA Marker DL2000; lanes 1, 3, 5, and 7 were control fragments of *actA*, *plcB*, and *orfX* and internal reference gene gyrB in NTSN, respectively, lanes 2, 4, 6, and 8 represented fragments of *actA*, *plcB*, *orfX* and internal reference gene *gyrB*, respectively, in NTSNΔ*actA*/*plcB*/*orfX*. **(B)** Western blot analysis of ActA and LLO expression by NTSN and NTSNΔ*actA*/*plcB*/*orfX*. M represents protein marker; lane 1 represented proteins ActA and LLO from NTSN; lane 2 represented ActA and LLO from NTSNΔ*actA*/*plcB*/*orfX*. **(C)** Phospholipase activity assay used YAC plate. NTSN and NTSNΔ*actA*/*plcB*/*orfX* bacterial colonies.

The positive clones were selected under erthyromycin and temperature selection pressures following the electroporation of pAULA *actA*-U/*orfX*-D into the competent parental strain NTSN. The deletion of all three genes were verified by RT-PCR with no amplification of the 3 fragments specific to *actA* (1,035 bp), *plcB* (511 bp), and *orfX* (302 bp) (Figure 1A). The deletion of *plcB* was further demonstrated by the loss of phospholipase activity on YAC medium, where a transparent zone was absent (Figure 1C). The knockout of *actA* was further verified by Western blotting, where the corresponding 97 kDa band was absent (Figure 1B). Taken together, these results confirmed successful construction of the NTSN*ΔactA/plcB/orfX* strain via homologous recombination technology.

### The Triple-Genes-Deletion Mutant Was Genetically Stabile

The genetic stability of the mutant strain was evaluated by serial passages. The genes *actA, plcB*, and *orfX* were unable to be detected at the 5th, 15th, and 30th generations indicating that the mutant strain was stable (Supplementary Figures S1A,B). Additionally, the genetic stability of the mutant strain was further determined by biochemical characterization. These results showed that the 5th, 15th, and 30th generations of the NTSN*ΔactA/plcB/orfX* strain had biochemical characteristics consistent with the first original generation, while lost the phospholipase activity comparison with the wild-type NTSN (Supplementary Table S1). In brief, these results indicated that the vaccine candidate was genetically stable.

### The Triple Genes Deletion Reduced *Lm* Virulence

**FIGURE 2:**
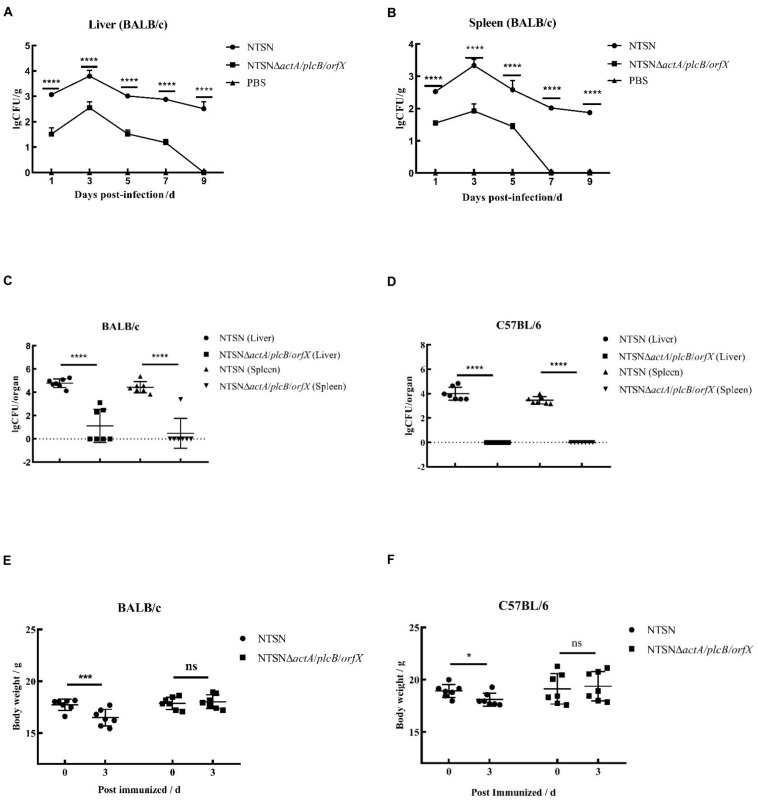
Bacterial loads and body weight changes in infected mice. Over time 6 mice in each group were abdominally injected with either NTSNΔ*actA*/*plcB*/*orfX* (5 × 10^5^ CFU), NTSN (5 × 10^3^ CFU), or PBS (150 mL). **(A)** Polyline with round black dots indicated the number of viable bacteria in the liver enumerated of the BALB/c mice infected by NTSN at 1, 3, 5, 7, and 9 days post-infection; While polyline with square black dots represented the BALB/c mice infected by NTSNΔ*actA*/*plcB*/*orfX*; And polyline with triangular black dots represented the BALB/c mice were injected by PBS. **(B)** Polyline with round black dots indicated the number of viable bacteria in the spleen enumerated of the BALB/c mice infected by NTSN at 1, 3, 5, 7, and 9 day post-infection; While polyline with square black dots represented the BALB/c mice were infected by NTSNΔ*actA*/*plcB*/*orfX*; And polyline with triangular black dots represented the BALB/c mice were injected by PBS. The log CFUs/g represented the mean of six mice per group. Each dot represented an organ from one infected mouse. Error bars represented SD, *n* = 3 independent experiments. Statistical analyses were carried out by Tukey’s multiple comparisons test: ****P < 0.0001. **(C)** The round black dots indicated to the bacterial load in the liver of BALB/c mice post NTSN infected 3 days; The square black dots indicated to the bacterial load in the liver of BALB/c mice post NTSNΔ*actA*/*plcB*/*orfX* infected 3 days; and the equilateral triangle black dots presented the bacterial load in the liver of BALB/c mice post NTSN infected 3 days; The inverted triangle black dots indicated to the bacterial load in the liver of BALB/c mice post NTSNΔ*actA*/*plcB*/*orfX* infected 3 days (Log CFU/organ). **(D)** The round black dot refers to the bacterial load in the liver of C57BL/6 mice post NTSN infected 3 days; The square black dots indicated the bacterial load in the spleen of C57BL/6 mice post NTSNΔ*actA*/*plcB*/*orfX infected* 3 days; and the equilateral triangle black dots indicated the bacterial load in the liver of C57BL/6 mice post NTSN infected 3 days; The inverted triangle black dots indicated the bacterial load in the spleen of C57BL/6 mice post NTSNΔ*actA*/*plcB*/*orfX* infected 3 days (Log CFU/organ). Body weight changes post infected mice at 3 days compared them with the pre-infection for statistical analysis. The log CFUs/organ represented the mean of seven mice per group. Each dot represents an organ from one infected mouse. Error bars represented SD, *n* = 3 independent experiments. Statistical analyses were carried out by Tukey’s multiple comparisons test: ****P < 0.0001. **(E)** Body weight changes of BALB/c mice post NTSN and NTSNΔ*actA*/*plcB*/*orfX* strain infection. **(F)** Body weight changes of BALB/c mice after NTSN and NTSNΔ*actA*/*plcB*/*orfX* strain infection. Statistical analyses were carried out by Sidak’s multiple comparisons test: ****P < 0.0001, ***P < 0.001, *P < 0.05, compared to the corresponding control group.

**FIGURE 3:**
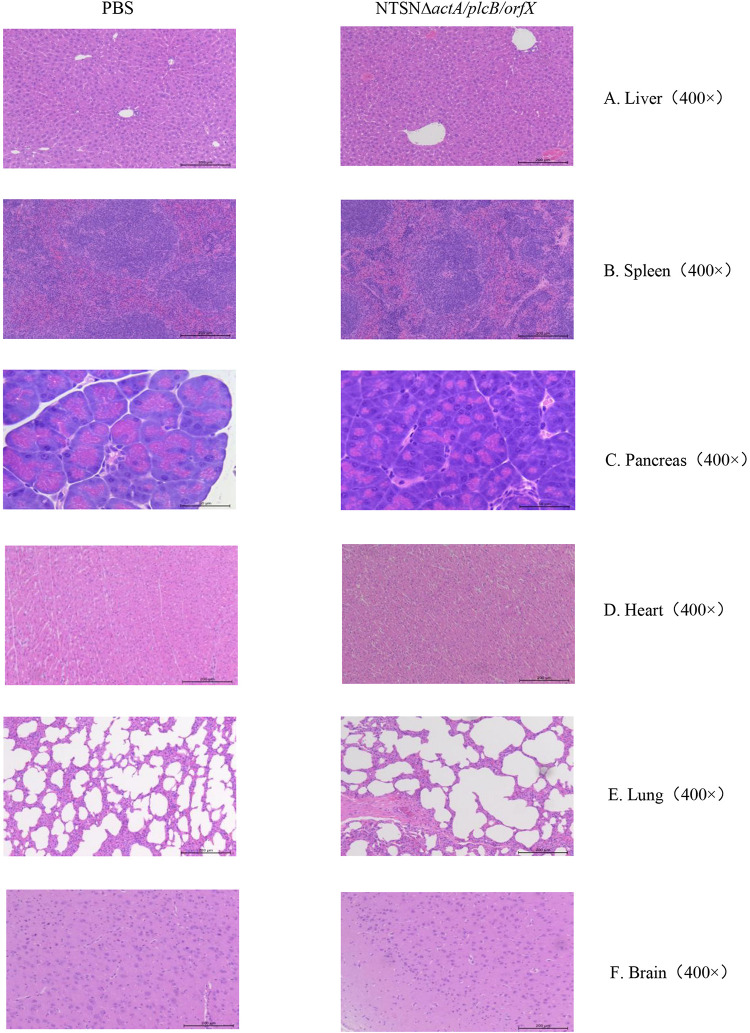
Histopathology of organs from mice injected with PBS or NTSNΔ*actA*/*plcB*/*orfX*. **(A)** Liver (400 ×). **(B)** Spleen (400 ×). **(C)** Pancreas (400 ×). **(D)** Heart (400 ×). **(E)** Lungs (400 ×). **(F)** Brain (400 ×).

The LD_50_ of the mutant strain was 794-folds higher than that of the wild-type *Lm* NTSN, indicated that its virulence in mice was remarkably decreased (Table 3). The virulence of the mutant strain was further evaluated by determining bacteria loads in organs of infected mice via intraperitoneal inoculation. Even an infection dose 100-folds higher than the parental strain resulted bacteria loads in spleen and liver significantly lower than the control group at each time point (*P* < 0.001). Additionally, the mutant was cleared by day 7 (spleen) or 9 (liver), suggesting a significant reduction in the colonization ability of the mutant strain (Figures 2A,B). Histopathology did not observe any pathological changes in the liver, spleen, heart, lungs, or brain of mice vaccinated with NTSNΔ*actA/plcB/orfX* (Figure 3), suggesting that the virulence of the mutant was dramatically reduced. It suggested that the virulence of the mutant was significantly reduced, and it had a certain potential safety in the application of humans and animals.

**TABLE 3:** The LD50 of NTSNΔ*actA*/*plcB*/*orfX* and NTSN.

**Strain**	**NTSN (1 × 10^4^)**			**NTSNΔ*actA*/*plcB*/*orfX* (1 × 10^7^)**		
Dose (CFU)	60	12	2.4	80	16	3.2
Mortality	4/5	4/5	2/5	4/5	4/5	4/5
LD50	6.31 × 10^4^			5.01 × 10^7^		

The susceptibility of *Lm* strains to BALB/c and C57BL/6 mice was compared with intraperitoneal infection at same dose. On day 3 post infection, the colonization ability of the triple-gene-deletion mutant in the spleen and liver either BALB/c and C57BL/6 mice were significantly lower than parental NTSN (*P* < 0.001) (Figures 2C,D). Body weight of BALB/c and C57BL/6 mice immunized with triple-genes-deletion group didn’t change (*P* > 0.05), while mice infected by parental NTSN significantly changed (*P* < 0.01, BALB/c mice; *P* < 0.05, C57BL/6 mice) (Figures 2E,F). This experiment verified that BALB/c mice were more susceptible to *Listeria* strains than C57BL/6 mice, and also demonstrated that the triple-gene-deletion mutant greatly reduced the ability to colonize and infect to two types of mice.

### A Strong Humoral Response Was Induced

**FIGURE 4:**
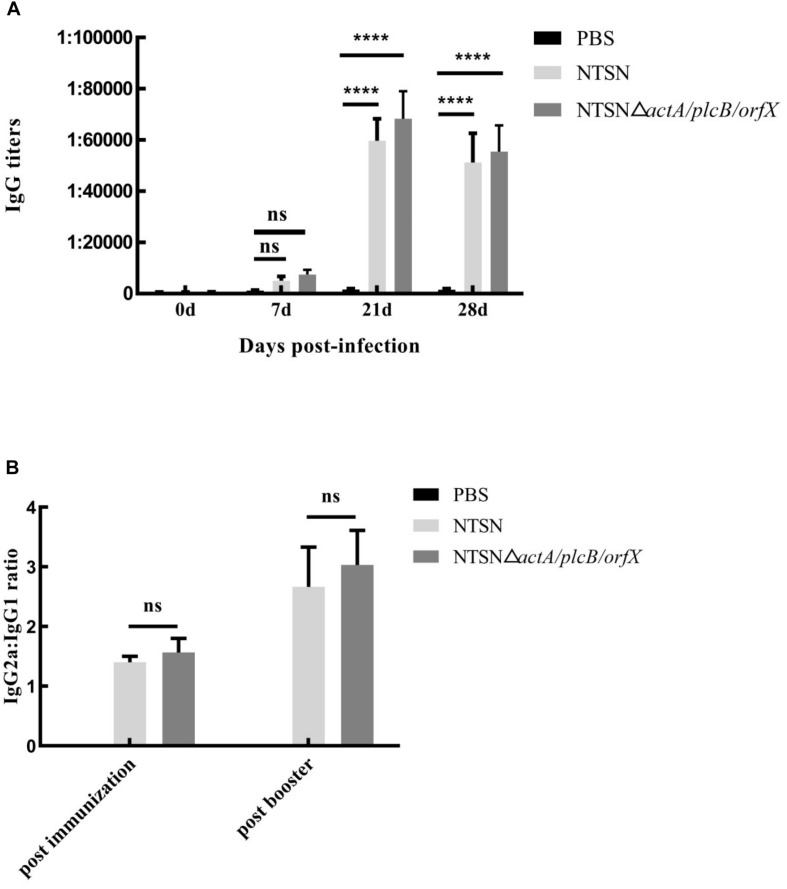
The levels of LLO antibodies in the sera of immunized mice. Mouse serum was harvested and the anti-LLO IgG antibody was detected by indirect ELISA. **(A)** IgG antibody in the serum of mice immunized with either PBS (black bar), NTSN (dark gray bar) or NTSNΔ*actA*/*plcB*/*orfX* (light gray bar) on days 7, 14, 21, and 28 post-immunization. **(B)** Determination of the antibody isotype profiles. The levels of IgG1 and IgG2a antibodies in the serum of PBS (black bar) NTSN (light gray bar), NTSNΔ*actA*/*plcB*/*orfX* (dark gray bar) infected groups on day 14 post-primary immunization and on day 14 post-booster immunization. Error bars represented SD, *n* = 3 independent experiments. Statistical analyses were carried out by Tukey’s multiple comparisons test: ****P < 0.0001, ns ≥ 0.05, compared to the corresponding control group.

We used an indirect ELISA to detect the presence of antibody against LLO in the serum of vaccinated mice. From the 7th day post-immunization, anti-LLO antibodies were significantly higher in mice immunized with the triple-genes-deletion mutant strain than those in the control group (7 days, *P* ≥ 0.05; 21 and 28 days, *P* < 0.001) (Figure 4A). Antibody levels increased successively and reached their highest levels at day 21 post-immunization. The above results indicated that the mutant strain stimulated a strong humoral immune response in mice.

To understand the biases of the immune response induced in mice vaccinated with the mutant strain, the IgG subtypes, including IgG1 and IgG2a, were determined by indirect ELISA and the ratio of IgG2a to IgG1 was calculated. The ratio of IgG2a/IgG1 exceeded 1 (Figure 4B) after the primary immunization. Importantly, the ratio of IgG2a/IgG1 increased to greater than 2 after the prime-boost immunization. This result confirmed that the NTSNΔ*actA/plcB/orfX* mutant strain induced a Th1 immune response in mice.

### Cellular Immune Response Was Elicited

**FIGURE 5:**
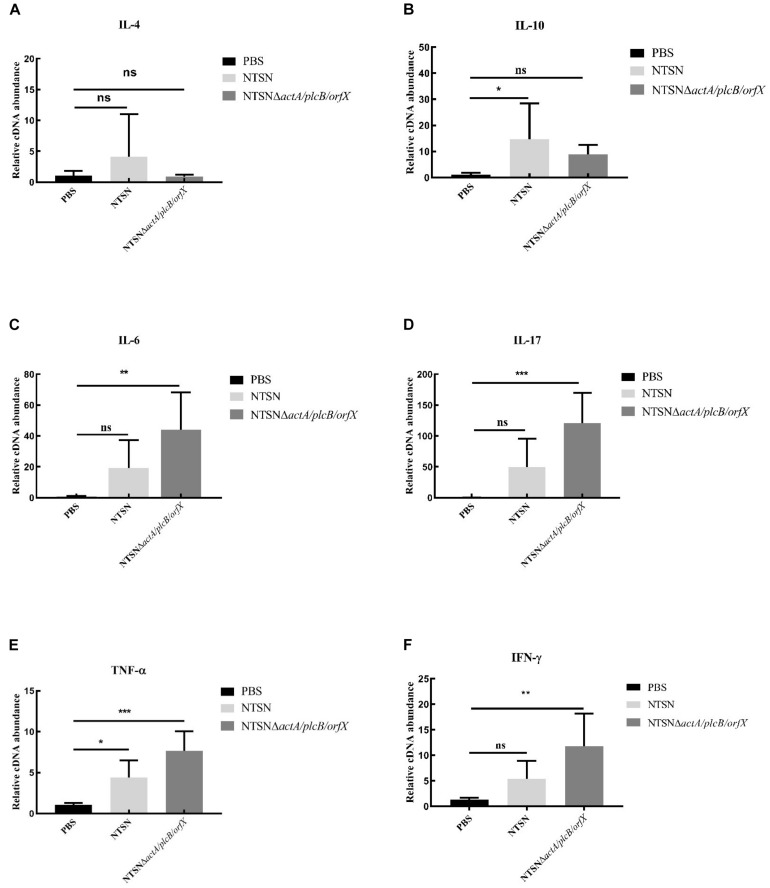
The Quantitative RT-PCR tested the expression level of inflammatory cytokines in the spleen tissue of immunized mice. The PBS control group (black bar), the NTSN immunized group (light gray bar), and the NTSNΔ*actA*/*plcB*/*orfX* group mice inflammatory cytokines (dark gray bar). **(A)** IL-4. **(B)** IL-6. **(C)** IL-10. **(D)** IL-17. **(E)** TNF-α. **(F)** IFN-γ (Change fold of expression level). Data presented were representative of three independent experiments. Error bars represented *SD*, *n* = 3 independent experiments. Statistical analyses were carried out by Tukey’s multiple comparisons test: ***P < 0.001, **P < 0.01, *P < 0.05, ns ≥ 05, compared to the control group.

The primary function of both CD4^+^ and CD8^+^ T cells is the secretion of proinflammatory cytokines IFN-γ, TNF-α, IL-6, etc. (Carpenter et al., 1994; Fan et al., 2015). The transcription levels of cytokines in the spleen of mice administration with *Listeria* strains were assessed by qRT-PCR. In Figure 5, qRT-PCR results showed that the transcription levels of IFN-γ, IL-17, TNF-α, IL-6, in NTSNΔ*actA/plcB/orfX* immunized mice were significant higher than control group (*P* < 0.01; *P* < 0.001). However, IL-4 and IL-10 transcription levels were no significant difference between the group immunized by the mutant strain and the control group (*P* > 0.05). These results indicated that triple-deletion mutant strain could induce a Th1 type immune response in mice. Our results suggested that this vaccine-candidate strain could elicit strong cellular immune response in mice, which enhanced the host’s immunity and defense against intracellular pathogens.

### The Vaccine Candidate Conferred Cross-Protection

**FIGURE 6:**
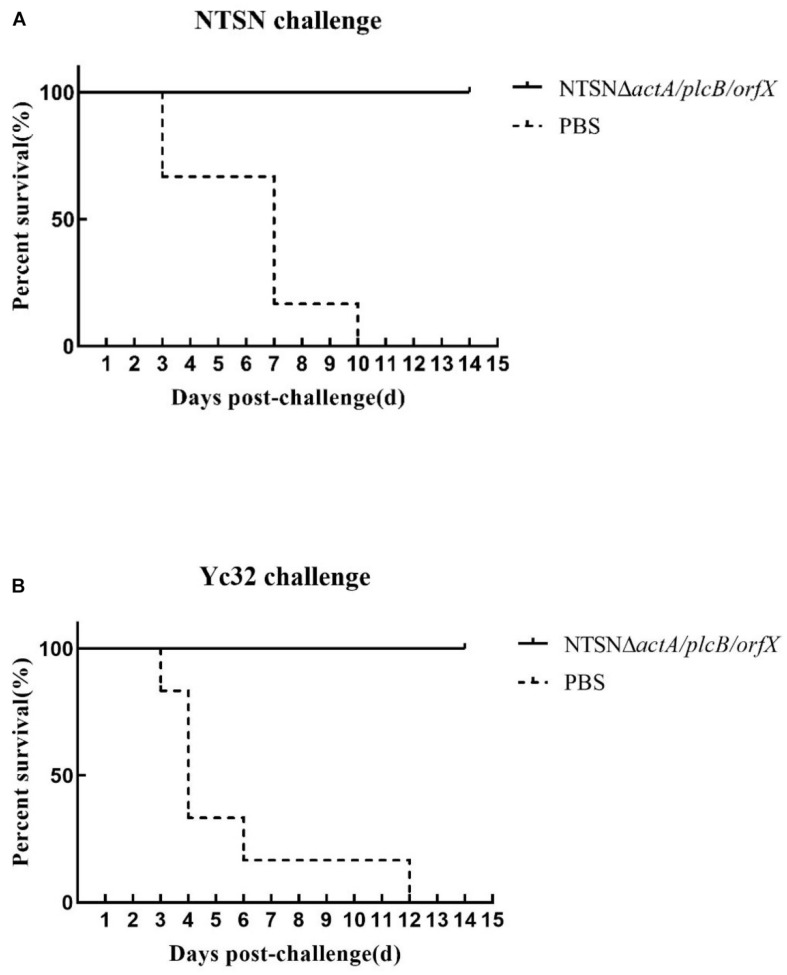
Survival of vaccinated mice challenged with NTSN and Yc32. **(A)** Solid line with circle icons indicated mice immunized with NTSNΔ*actA*/*plcB*/*orfX* and challenged by wild-type NTSN; Dotted line with square icons represented mice immunized with NTSNΔ*actA*/*plcB*/*orfX* and challenged by wild-type Yc32. **(B)** Dotted line with rhombus icons represented mice inoculated with PBS and challenged by wild-type NTSN; Short horizontal line with triangle icons indicated mice inoculated with PBS and challenged with wild-type Yc32. Survival curves of mice (*n* = 6/group) inoculated intragastrically with 7 × 10^6^ CFU of NTSN and 2.5 × 10^7^ CFU of Yc32, respectively, and observed continuously for 14 days. The data represented two independent experiments.

The protective efficacy of NTSNΔ*actA/plcB/orfX* in mice was evaluated by challenges with either the wild-type strain, or 1/2b strain Yc32. As shown in Figure 6, vaccination with NTSNΔ*actA/plcB/orfX* provided 100% protection against challenges with either the serovar 4b strain NTSN or 1/2b strain Yc32. However, the PBS control vaccination group provided 0% protection against challenges by either serovar 4b or 1/2b strain (Figure 6). These results indicated that the vaccine-candidate strain NTSNΔ*actA/plcB/orfX* could provide cross-protective immunity to mice against challenges with either serovar 4b or 1/2b strain.

## Discussion

In 1881, Pasteur invented an attenuated anthrax vaccine against *Bacillus anthracis*, which has been the basis of the classic vaccine ever since. *Lm* is one of the most deadly foodborne pathogen, causing listeriosis. Vaccine immunization is one of the most effective strategy to prevent listeriosis infections. Researchers have developed a diverse array of *Listeria* vaccine platforms, e.g., *Listeria* live attenuated vaccine (McLaughlin et al., 2013), live vector vaccine (Yin et al., 2017; Zeng et al., 2020), inactivated vaccine (Jazani et al., 2010), inactivated but metabolically active (KBMA) vaccine (Lauer et al., 2008), irradiated vaccine (Dong et al., 2009), Bacterial ghosts (BGs) (Wu et al., 2017), subunit vaccine (Ansari et al., 2012; Rodriguez-Del Rio et al., 2015), nano-vaccine (Calderon-Gonzalez et al., 2017), and peptides-loaded DC vaccine (Kono et al., 2012; Jensen et al., 2013). However, there is not yet a *Listeria* vaccine that has been approved by the FDA department on the market (Cory and Chu, 2014). Subunit vaccines and inactivated vaccines have the advantage of high safety, whereas they induce limited immunogenicity, particularly, cellular immune response (Ansari et al., 2012; Luo and Cai, 2012; Cheng et al., 2014). Live attenuated *Lm* is considered as a promising vaccine vector (McLaughlin et al., 2013), the clinical applications of *Listeria-*vectored vaccines have a bright future (Maciag et al., 2009). At the same time, the potential risk to immunodeficient population (Kono et al., 2012) must not be overlooked. Therefore, we have focused on the safety of live attenuated vaccines. In this study, we constructed a triple-virulence-genes deletion vaccine strain with homologous recombination and tested its safety in a murine infection model. The safety of the vaccine strain increased 794-folds above its parental strain. Additionally, it was completely eliminated at day 9 post-immunization with the doses 100-times higher than the LD_50_, and there was no obvious pathological change in any organ. In brief, the safety of the mutant strain was dramatically improved.

*Lm* has a unique intracellular lifecycle, which escapes from phagocytosis by host macrophages, whereby it enters the host cytoplasm and replicates (Pizarro-Cerda and Cossart, 2018). An efficient adaptive immune response is required to provide protective immunity, thus the ideal *Listeria* vaccine should effectively stimulate both cellular and humoral immune responses. Cytokines play an important role in the host’s activation of the immune system (Zharkova et al., 2017), i.e., pro-inflammatory cytokines IFN-γ and TNF-α, which have major roles in inducing Th1 immune responses to protect against cellular pathogen (Allie et al., 2013; Romagnoli et al., 2017). IL-17 is a strong pro-inflammatory factor which is related with multiple disease, involved in inducing cellular immune responses (Amatya et al., 2017). And the IL-6 is produced to promote T cell differentiation, regulate cellular immune response, and clear the intracellular *Lm* (Hoge et al., 2013). Our results showed that significantly higher expression levels of pro-inflammatory cytokines IFN-γ, TNF-α, IL-10, and IL-17 were elicited post-immunization with the vaccine candidate, while the levels of anti-inflammatory cytokine IL-4 and IL-10 were unchanged compared with the control group. IL-10 is one of the most important anti-inflammatory cytokines generated during infectious diseases (Peñaloza et al., 2018). This mutant vaccine candidate increased levels of IFN-γ, IL-17, IL-6, and TNF-α, along with decreased levels of IL-4, IL-10 lead to the differentiation of helper T cells into the Th1 type (Haring et al., 2005; Tan et al., 2018). Thus our results suggest that our vaccine candidate induces Th1-type cellular response, contributes to the host response to the intracellular bacteria, and ultimately improves the cellular immune response of mice to eliminate the intracellular pathogen *Lm*.

Increasing evidence suggests that humoral immunity is important to defend against intracellular pathogens (Mahdy et al., 2019; Lew et al., 2020). Recent evidence suggests that the antibody response to *Mycobacterium tuberculosis* (*M.tb*) antigens may play a more significant role in impairing the extra-pulmonary dissipation of *M.tb* and antibody-mediated enhancement of mycobacteria phagocytosis by macrophages. Thus, it contributes to control intracellular *M.tb* (Lu et al., 2016). *Lm* is a gastrointestinal pathogen, thus the characterization of humoral immunity elicited by this attenuated vaccine was explored. In our results, the anti-LLO antibodies were significantly higher in the vaccine-candidate group, and peaked on day 21. Listerolysin O (LLO) harbors a variety of B and T cell epitopes and is an important protective antigen against *Lm*. Our results suggest that candidate vaccine strain could induce a strong humoral immune response to LLO. IgG2a and IgG1 are used as indicators for Th1 and Th2 responses, respectively (Tao et al., 2017), thus the vaccine-induced T-cell phenotype can be evaluated by the ratio of IgG2a/IgG1. Our results verified that vaccination significantly increased the levels of serum IgG2a, and decreased IgG1 levels, such that the ratio of IgG2a/IgG1 was more than 2 after the booster immunization. This further confirms that the vaccine candidate NTSNΔ*actA*/*plcB*/*orfX* can induce both humoral and cellular immune responses in mice.

According the characterization of the somatic and flagellar antigens, *Lm* strains are classified to 14 serotypes, of which serotype 4b strain is responsible for the majority of recorded, invasive listeriosis outbreaks, followed by the 1/2b and 1/2a serotypes (Wiedmann et al., 1996; Walecka-Zacharska et al., 2013). Linde et al. (1995) developed an attenuated bivalent (1/2a and 4b) vaccine by metabolic drift mutation, which afforded 95% protection in mice against a lethal dose of the parental strain. However, a vaccine against the serotype 4b and 1/2b strains infection has not been reported. Our study shows that the vaccine not only affords 100% immunoprotection against the serotype 4b strain in mice, but also 100% protection against the 1/2b strain. These two strains, 4b and 1/2b, have the same flagellum antigen b and different somatic antigens, which are 4 and 1, respectively (Liu, 2006). If the common flagella antigens playing crucial role in cross-protective immunity needs to be further elucidated. Our cross-protective vaccine against 4b and 1/2b serotypes provides a new strategy to prevent and control listeriosis.

## Conclusion

In conclusion, the highly attenuated *Lm* vaccine strain NTSNΔ*actA*/*plcB*/*orfX* has high safety and induces significant cellular and humoral immune response. It can not only afford protective immunity against serotype 4b strain challenges, but also serotype 1/2b strain, thus prevent infection by multiple pathogenic serotypes of *Lm*. Therefore, this vaccine strain is a potential candidate for controlling *Listeria* infection in human.

## Data Availability Statement

The raw data supporting the conclusions of this article will be made available by the authors, without undue reservation, to any qualified researcher.

## Ethics Statement

The animal study was reviewed and approved by the Institutional Animal Ethics Committee of Yangzhou University.

## Author Contributions

YY and XJ designed the experiments. TZ and FM performed the experiments and analyzed the results. HY, ZL, YF, GL, JL, and XS were involved in animal experiments. JC and CM were involved in immune related experiments. YY and FM wrote the manuscript. All authors read and approved the final manuscript.

## Conflict of Interest

The authors declare that the research was conducted in the absence of any commercial or financial relationships that could be construed as a potential conflict of interest.

## References

[B1] Allie N. Grivennikov S. I. Keeton R. Hsu N. J. Bourigault M. L. Court N. (2013). Prominent role for T cell-derived tumour necrosis factor for sustained control of *Mycobacterium tuberculosis* infection. *Sci*. *Rep*. 3:1809. 10.1038/srep01809 23657146 PMC3648802

[B2] Amatya N. Garg A. V. Gaffen S. L. (2017). IL-17 signaling: the yin and the yang. *Trends Immunology.* 38 310–322. 10.1016/j.it.2017.01.006 28254169 PMC5411326

[B3] Angelo K. M. Conrad A. R. Saupe A. Dragoo H. West N. Sorenson A. (2017). Multistate outbreak of *Listeria monocytogenes* infections linked to whole apples used in commercially produced, prepackaged caramel apples: United States, 2014-2015. *Epidemiol. Infect.* 145 848–856. 10.1017/S0950268816003083 28065170 PMC6542465

[B4] Ansari M. A. Zubair S. Tufail S. Ahmad E. Khan M. R. Quadri Z. (2012). Ether lipid vesicle-based antigens impart protection against experimental listeriosis. *Int. J. Nanomed.* 7 2433–2447. 10.2147/IJN.S25875 22745536 PMC3383290

[B5] Berg R. E. Crossley E. Murray S. Forman J. (2005). Relative contributions of NK and CD8 T cells to IFN-gamma mediated innate immune protection against *Listeria monocytogenes*. *J. Immunol.* 175 1751–1757. 10.4049/jimmunol.175.3.1751 16034116 PMC1615713

[B6] Boneca I. G. Dussurget O. Cabanes D. Nahori M. A. Sousa S. Lecuit M. (2007). A critical role for peptidoglycan N-deacetylation in *Listeria* evasion from the host innate immune system. *Proc. Natl. Acad. Sci. U S A* 104 997–1002. 10.1073/pnas.0609672104 17215377 PMC1766339

[B7] Buchanan R. L. Gorris L. G. M. Hayman M. M. Jackson T. C. Whiting R. C. (2017). A review of *Listeria monocytogenes*: an update on outbreaks, virulence, dose-response, ecology, and risk assessments. *Food Control* 75 1–13. 10.1016/j.foodcont.2016.12.016

[B8] Calderon-Gonzalez R. Frande-Cabanes E. Teran-Navarro H. Marimon J. Freire J. Salcines-Cuevas D. (2017). GAPDH1-22 nanovaccines prevent neonatal listeriosis by blocking microglial apoptosis and bacterial dissemination. *Oncotarget* 8 53916–53934. 10.18632/oncotarget.19405 28903312 PMC5589551

[B9] Camargo A. C. de Castilho N. P. da Silva D. A. Vallim D. C. Hofer E. Nero L. A. (2015). Antibiotic resistance of *Listeria monocytogenes* isolated from meat-processing environments, beef products, and clinical cases in Brazil. *Microb. Drug Resist.* 21 458–462. 10.1089/mdr.2014.0270 25756759

[B10] Carpenter E. A. Ruby J. Ramshaw I. A. (1994). IFN-γ, TNF, and IL-6 production by vaccinia virus immune spleen cells: An in vitro study. *J. Immunol.* 152 2652–2659. 8144873

[B11] Chamoun M. N. Blumenthal A. Sullivan M. J. Schembri M. A. Ulett G. C. (2018). Bacterial pathogenesis and interleukin-17: interconnecting mechanisms of immune regulation, host genetics, and microbial virulence that influence severity of infection. *Crit. Rev. Microbiol.* 44 465–486. 10.1080/1040841X.2018.1426556 29345518

[B12] Cheng W. K. Wee K. Kollmann T. R. Dutz J. P. Pasetti M. F. (2014). Topical CpG adjuvantation of a protein-based vaccine induces protective immunity to *Listeria monocytogenes*. *Clin. Vaccin. Immunol.* 21 329–339. 10.1128/cvi.00734-13 24391136 PMC3957669

[B13] Cory L. Chu C. (2014). ADXS-HPV: a therapeutic *Listeria* vaccination targeting cervical cancers expressing the HPV E7 antigen. *Hum. Vaccin. Immunother.* 10 3190–3195. 10.4161/hv.34378 25483687 PMC4514130

[B14] Crittenden M. Bahjat K. S. Li R. Gore P. Fountain C. Hanson B. (2015). Phase I study of safety and immunogenicity of ADU-623, a live-attenuated *Listeria monocytogenes* vaccine (ΔactA/ΔinlB) expressing EGFRVIII and NY-ESO-1, in patients with who grade III/IV astrocytomas. *J. Immu. Ther. Cancer* 3:162 10.1186/2051-1426-3-s2-p162

[B15] Czuprynski C. J. Faith N. G. Steinberg H. (2002). Ability of the *Listeria monocytogenes* strain Scott A to cause systemic infection in mice infected by the intragastric route. *Appl. Environ. Microbiol.* 68 2893–2900. 10.1128/aem.68.6.2893-2900.2002 12039747 PMC123921

[B16] de las Heras A. Cain R. J. Bielecka M. K. Vazquez-Boland J. A. (2011). Regulation of *Listeria* virulence: PrfA master and commander. *Curr. Opin. Microbiol.* 14 118–127. 10.1016/j.mib.2011.01.005 21388862

[B17] Ding C. Liu Q. Li J. Ma J. Wang S. Dong Q. (2019). Attenuated *Listeria monocytogenes* protecting zebrafish (Danio rerio) against *Vibrio species* challenge. *Microb. Pathog.* 132 38–44. 10.1016/j.micpath.2019.03.040 30986451

[B18] Dong H. Jiao X. A. Pan Z. Yin Y. Liu S. (2009). Immunogenicity comparision of *Listeria monocytogenes* inactivated by gamma-irradiation or traditional treatments. *Wei Sheng Wu Xue Bao* 49 269–273. 19445186

[B19] D’Orazio S. E. F. (2019). Innate and adaptive immune responses during *Listeria monocytogenes* infection. *Microbiol. Spectr* 7 G3–G0065. 10.1128/microbiolspec.gpp3-0065-2019 PMC11086964 31124430

[B20] Duranti A. Sabbatucci M. Blasi G. Acciari V. A. Ancora M. Bella A. (2018). A severe outbreak of listeriosis in central Italy with a rare pulsotype associated with processed pork products. *J. Med. Microbiol.* 67 1351–1360. 10.1099/jmm.0.000785 30024370

[B21] Fan D. Li W. Yang Y. Zhang X. Zhang Q. Yan Y. (2015). Redirection of CD4+ and CD8+ T lymphocytes via an anti-CD3 x anti-CD19 bi-specific antibody combined with cytosine arabinoside and the efficient lysis of patient-derived B-ALL cells. *J. Hematol. Oncol.* 8:108. 10.1186/s13045-015-0205-6 26444983 PMC4596481

[B22] Freches D. Korf H. Denis O. Havaux X. Huygen K. Romano M. (2013). Mice genetically inactivated in interleukin-17A receptor are defective in long-term control of *Mycobacterium tuberculosis* infection. *Immunology* 140 220–231. 10.1111/imm.12130 23721367 PMC3784168

[B23] Haring J. S. Badovinac V. P. Olson M. R. Varga S. M. Harty J. T. (2005). In vivo generation of pathogen-specific Th1 cells in the absence of the IFN-gamma receptor. *J. Immunol.* 175 3117–3122. 10.4049/jimmunol.175.5.3117 16116201

[B24] Hernandez-Milian A. Payeras-Cifre A. (2014). What is new in listeriosis? *Biomed. Res. Int.* 2014 358051. 10.1155/2014/358051 24822197 PMC4005144

[B25] Hoge J. Yan I. Janner N. Schumacher V. Chalaris A. Steinmetz O. M. (2013). IL-6 controls the innate immune response against *Listeria monocytogenes* via classical IL-6 signaling. *J. Immunol.* 190 703–711. 10.4049/jimmunol.1201044 23241882

[B26] Ito D. Nojima S. Nishide M. Okuno T. Takamatsu H. Kang S. (2015). mTOR complex signaling through the SEMA4A-Plexin B2 axis is required for optimal activation and differentiation of CD8+ T cells. *J. Immunol.* 195 934–943. 10.4049/jimmunol.1403038 26116513 PMC4505953

[B27] Jahan M. Holley R. A. (2016). Transfer of antibiotic resistance from *Enterococcus faecium* of fermented meat origin to *Listeria monocytogenes* and *Listeria innocua*. *Lett. Appl. Microbiol.* 62 304–310. 10.1111/lam.12553 26854329

[B28] Jamshidi A. Zeinali T. (2019). Significance and characteristics of *Listeria monocytogenes* in poultry products. *Int. J. Food. Sci.* 2019 7835253. 10.1155/2019/7835253 31139641 PMC6500651

[B29] Jazani N. H. Karimzad M. Mazloomi E. Sohrabpour M. Hassan Z. M. Ghasemnejad H. (2010). Evaluation of the adjuvant activity of naloxone, an opioid receptor antagonist, in combination with heat-killed *Listeria monocytogenes* vaccine. *Microbes Infect.* 12 382–388. 10.1016/j.micinf.2010.02.001 20152926

[B30] Jensen S. Steffensen M. A. Jensen B. A. Schluter D. Christensen J. P. Thomsen A. R. (2013). Adenovirus-based vaccine against *Listeria monocytogenes*: extending the concept of invariant chain linkage. *J. Immunol.* 191 4152–4164. 10.4049/jimmunol.1301290 24043891

[B31] Johnson P. V. Blair B. M. Zeller S. Kotton C. N. Hohmann E. L. (2011). Attenuated *Listeria monocytogenes* vaccine vectors expressing influenza a nucleoprotein: preclinical evaluation and oral inoculation of volunteers. *Microbiol. Immunol.* 55 304–317. 10.1111/j.1348-0421.2011.00322.x 21338384 PMC3082616

[B32] Kathariou S. Graves L. Buchrieser C. Glaser P. Siletzky R. M. Swaminathan B. (2006). Involvement of closely related strains of a new clonal group of *Listeria monocytogenes* in the 1998-99 and 2002 multistate outbreaks of foodborne listeriosis in the United States. *Foodborne Pathog. Dis.* 3 292–302. 10.1089/fpd.2006.3.292 16972778

[B33] Kono M. Nakamura Y. Suda T. Uchijima M. Tsujimura K. Nagata T. (2012). Enhancement of protective immunity against intracellular bacteria using type-1 polarized dendritic cell (DC) vaccine. *Vaccine* 30 2633–2639. 10.1016/j.vaccine.2012.02.026 22365841

[B34] Lauer P. Hanson B. Lemmens E. E. Liu W. Luckett W. S. Leong M. L. (2008). Constitutive activation of the PrfA regulon enhances the potency of vaccines based on live-attenuated and killed but metabolically active *Listeria monocytogenes* strains. *Infect. Immun.* 76 3742–3753. 10.1128/IAI.00390-08 18541651 PMC2493200

[B35] Lew M. H. Norazmi M. N. Tye G. J. (2020). Enhancement of immune response against *Mycobacterium tuberculosis* HspX antigen by incorporation of combined molecular adjuvant (CASAC). *Mol. Immunol.* 117 54–64. 10.1016/j.molimm.2019.10.023 31739193

[B36] Lin P. L. Flynn J. L. (2015). CD8 T cells and *Mycobacterium tuberculosis* infection. *Semin. Immunopathol.* 37 239–249. 10.1007/s00281-015-0490-8 25917388 PMC4439333

[B37] Linde K. Fthenakist G. C. Lippmannz R. Kinneg J. Abraham A. (1995). The efficacy of a live *Listeria monocytogenes* combined serotype 1/2a and serotype 4b vaccine. *Vaccine* 13 923–926. 10.1016/0264-410x(95)00010-x 7483765

[B38] Liu D. (2006). Identification, subtyping and virulence determination of *Listeria monocytogenes*, an important foodborne pathogen. *J. Med. Microbiol.* 55(Pt 6), 645–659. 10.1099/jmm.0.46495-0 16687581

[B39] Lu L. L. Chung A. W. Rosebrock T. R. Ghebremichael M. Yu W. H. Grace P. S. (2016). A functional role for antibodies in tuberculosis. *Cell* 167 433–443e414. 10.1016/j.cell.2016.08.072 27667685 PMC5526202

[B40] Luo X. Cai X. (2012). A combined use of Autolysin p60 and Listeriolysin O antigens induces high protective immune responses against *Listeria monocytogenes* infection. *Curr. Microbiol.* 65 813–818. 10.1007/s00284-012-0238-9 23001425

[B41] Maciag P. C. Radulovic S. Rothman J. (2009). The first clinical use of a live-attenuated *Listeria monocytogenes* vaccine: a phase I safety study of Lm-LLO-E7 in patients with advanced carcinoma of the cervix. *Vaccine* 27 3975–3983. 10.1016/j.vaccine.2009.04.041 19389451

[B42] Mahdy S. E. Liu S. Su L. Zhang X. Chen H. Pei X. (2019). Expression of the VP1 protein of FMDV integrated chromosomally with mutant *Listeria monocytogenes* strain induced both humoral and cellular immune responses. *Appl. Microbiol. Biotechnol.* 103 1919–1929. 10.1007/s00253-018-09605-x 30627793

[B43] McLaughlin H. P. Bahey-El-Din M. Casey P. G. Hill C. Gahan C. G. M. (2013). A mutant in the *Listeria monocytogenes* fur-regulated virulence locus (frvA) induces cellular immunity and confers protection against listeriosis in mice. *J. Med. Microbiol.* 62(Pt 2), 185–190. 10.1099/jmm.0.049114-0 23105022

[B44] Miya S. Kimura B. Sato M. Takahashi H. Ishikawa T. Suda T. (2008). Development of a multilocus variable-number of tandem repeat typing method for *Listeria monocytogenes* serotype 4b strains. *Int. J. Food. Microbiol.* 124 239–249. 10.1016/j.ijfoodmicro.2008.03.023 18457891

[B45] Mostowy S. Cossart P. (2012). Virulence factors that modulate the cell biology of *Listeria* infection and the host response. *Adv. Immunol.* 113 19–32. 10.1016/B978-0-12-394590-7.00007-5 22244577

[B46] Murai M. Turovskaya O. Kim G. Madan R. Karp C. L. Cheroutre H. (2009). Interleukin 10 acts on regulatory T cells to maintain expression of the transcription factor Foxp3 and suppressive function in mice with colitis. *Nat. Immunol.* 10 1178–1184. 10.1038/ni.1791 19783988 PMC2898179

[B47] Murray E. G. D. Webb R. A. Swann M. B. (2005). A disease of rabbits characterised by a large mononuclear leucocytosis, caused by a hitherto undescribed bacillus *Bacterium monocytogenes* (n.sp.). *J. Pathol. Bacteriol.* 29 407–439. 10.1002/path.1700290409

[B48] Nguyen B. N. Portnoy D. A. (2020). An inducible cre-lox system to analyze the role of LLO in *Listeria monocytogenes* pathogenesis. *Toxins* 12:38. 10.3390/toxins12010038 31936068 PMC7020405

[B49] Nomura T. Kawamura I. Tsuchiya K. Kohda C. Baba H. Ito Y. (2002). Essential role of interleukin-12 (IL-12) and IL-18 for gamma interferon production induced by listeriolysin O in mouse spleen cells. *Infect. Immun.* 70 1049–1055. 10.1128/iai.70.3.1049-1055.2002 11854182 PMC127750

[B50] Opitz B. Puschel A. Beermann W. Hocke A. C. Forster S. Schmeck B. (2006). *Listeria monocytogenes* activated p38 MAPK and induced IL-8 secretion in a nucleotide-binding oligomerization domain 1-dependent manner in endothelial cells. *J. Immunol.* 176 484–490. 10.4049/jimmunol.176.1.484 16365441

[B51] Peñaloza H. F. Noguera L. P. Riedel C. A. Bueno S. M. (2018). Expanding the current knowledge about the role of Interleukin-10 to major concerning bacteria. *Front. Microbiol.* 9:2047. 10.3389/fmicb.2018.02047 30279680 PMC6153308

[B52] Pizarro-Cerda J. Cossart P. (2018). *Listeria monocytogenes*: cell biology of invasion and intracellular growth. *Microbiol. Spectr.* 6 G3–G0013. 10.1128/microbiolspec.GPP3-0013-2018 30523778 PMC11633638

[B53] Rodriguez-Del Rio E. Marradi M. Calderon-Gonzalez R. Frande-Cabanes E. Penades S. Petrovsky N. (2015). A gold glyco-nanoparticle carrying a Listeriolysin O peptide and formulated with Advax delta inulin adjuvant induces robust T-cell protection against listeria infection. *Vaccine* 33 1465–1473. 10.1016/j.vaccine.2015.01.062 25659269

[B54] Rolhion N. Cossart P. (2017). How the study of *Listeria monocytogenes* has led to new concepts in biology. *Fut. Microbiol*. 12 621–638. 10.2217/fmb-2016-0221 28604108

[B55] Romagnoli P. A. Fu H. H. Qiu Z. Khairallah C. Pham Q. M. Puddington L. (2017). Differentiation of distinct long-lived memory CD4 T cells in intestinal tissues after oral *Listeria monocytogenes* infection. *Mucosal Immunol.* 10 520–530. 10.1038/mi.2016.66 27461178 PMC5272904

[B56] Saklani-Jusforgues H. Fontan E. Soussi N. Milon G. Goossens P. L. (2003). Enteral immunization with attenuated recombinant *Listeria monocytogenes* as a live vaccine vector: organ-dependent dynamics of CD4 T lymphocytes reactive to a *Leishmania* major tracer epitope. *Infect. Immun.* 71 1083–1090. 10.1128/iai.71.3.1083-1090.2003 12595418 PMC148854

[B57] Schutte C. M. Van der Meyden C. H. Kakaza M. Lockhat Z. Van der Walt E. (2019). Life-threatening *Listeria* meningitis: Need for revision of South African acute bacterial meningitis treatment guidelines. *S. Afr. Med. J.* 109 296–298. 10.7196/SAMJ.2019.v109i5.13866 31131793

[B58] Smith A. M. Tau N. P. Smouse S. L. Allam M. Ismail A. Ramalwa N. R. (2019). Outbreak of *Listeria monocytogenes* in South Africa, 2017-2018: laboratory activities and experiences associated with whole-genome sequencing analysis of isolates. *Foodborne Pathog. Dis.* 16 524–530. 10.1089/fpd.2018.2586 31062992 PMC6653791

[B59] Stockinger S. Reutterer B. Schaljo B. Schellack C. Brunner S. Materna T. (2004). IFN regulatory factor 3-dependent induction of type I IFNs by intracellular bacteria is mediated by a TLR- and Nod2-independent mechanism. *J. Immunol.* 173 7416–7425. 10.4049/jimmunol.173.12.7416 15585867

[B60] Tan C. L. Peluso M. J. Drijvers J. M. Mera C. M. Grande S. M. Brown K. E. (2018). CD160 stimulates CD8+ T cell responses and is required for optimal protective immunity to *Listeria monocytogenes*. *ImmunoHorizons* 2 238–250. 10.4049/immunohorizons.1800039 31022694 PMC7464592

[B61] Tao W. Fu T. He Z. Hu R. Jia L. Hong Y. (2017). Evaluation of immunostimulatory effects of n-(2-hydroxy) propyl-3-trimethylammonium chitosan chloride for improving live attenuated hepatitis a virus vaccine efficacy. *Viral. Immunol.* 30 120–126. 10.1089/vim.2016.0099 27918250

[B62] Thompson R. J. Bouwer H. G. Portnoy D. A. Frankel F. R. (1998). Pathogenicity and immunogenicity of a *Listeria monocytogenes* strain that requires D-alanine for growth. *Infect. Immun.* 66 3552–3561. 10.1128/iai.66.8.3552-3561.1998 9673233 PMC108386

[B63] Vasanthakrishnan R. B. de Las Heras A. Scortti M. Deshayes C. Colegrave N. Vazquez-Boland J. A. (2015). PrfA regulation offsets the cost of *Listeria* virulence outside the host. *Environ. Microbiol.* 17 4566–4579. 10.1111/1462-2920.12980 26178789 PMC4737189

[B64] Walecka-Zacharska E. Kosek-Paszkowska K. Bania J. Karpiskova R. Stefaniak T. (2013). Salt stress-induced invasiveness of major *Listeria monocytogenes* serotypes. *Lett. Appl. Microbiol.* 56 216–221. 10.1111/lam.12036 23294476

[B65] Wiedmann M. Bruce J. L. Knorr R. Bodis M. Cole E. M. McDowell C. I. (1996). Ribotype diversity of *Listeria monocytogenes* strains associated with outbreaks of listeriosis in ruminants. *J. Clin. Microbiol.* 34 1086–1090. 10.1128/jcm.34.5.1086-1090.1996 8727881 PMC228960

[B66] Wu X. Ju X. Du L. Yuan J. Wang L. He R. (2017). Production of bacterial ghosts from gram-positive pathogen *Listeria monocytogenes*. *Foodborne Pathog. Dis.* 14 1–7. 10.1089/fpd.2016.2184 27982711

[B67] Yao H. Kang M. Wang Y. Feng Y. Kong S. Cai X. (2018). An essential role for hfq involved in biofilm formation and virulence in serotype 4b *Listeria monocytogenes*. *Microbiol. Res.* 215 148–154. 10.1016/j.micres.2018.07.001 30172301

[B68] Yeung P. S. M. Zagorski N. Marquis H. (2005). The metalloprotease of *Listeria monocytogenes* controls cell wall translocation of the broad-range phospholipase C. *J. Bacteriol.* 187 2601–2608. 15805506 10.1128/JB.187.8.2601-2608.2005 PMC1070396

[B69] Yin Y. Lian K. Zhao D. Tao C. Chen X. Tan W. (2017). A promising *Listeria*-vectored vaccine induces Th1-type immune responses and confers protection against tuberculosis. *Front. Cell Infect. Microbiol.* 7:407. 10.3389/fcimb.2017.00407 29034213 PMC5626977

[B70] Yin Y. Yao H. Doijad S. Kong S. Shen Y. Cai X. (2019). A hybrid sub-lineage of *Listeria monocytogenes* comprising hypervirulent isolates. *Nat. Commun.* 10:4283. 10.1038/s41467-019-12072-1 31570766 PMC6768887

[B71] Yin Y. Zhu G. Geng S. Hu M. Jiao X. (2008). [Construction and characterization of a mutant strain of *Listeria monocytogenes* with a deletion of actA and plcB]. *Wei Sheng Wu Xue Bao* 48 299–303. 18479054

[B72] Zeng H. Xie M. Ding C. Ma J. Xu D. Wang X. (2020). Attenuated *Listeria monocytogenes* as a vaccine vector for the delivery of OMPW, the outer membrane protein of *Aeromonas hydrophila*. *Front. Microbiol.* 11:70. 10.3389/fmicb.2020.00070 32153514 PMC7047129

[B73] Zharkova O. Celhar T. Cravens P. D. Satterthwaite A. B. Fairhurst A. M. Davis L. S. (2017). Pathways leading to an immunological disease: systemic lupus erythematosus. *Rheumatology* 56 i55–i66. 10.1093/rheumatology/kew427 28375453 PMC5410978

