# Supplementary material for: A Cross-Protective Vaccine Against 4b and 1/2b Listeria monocytogenes
Source: Front Microbiol. 2020 Dec 11;11:569544. doi: 10.3389/fmicb.2020.569544 (PMC7759533; doi:10.3389/fmicb.2020.569544)
Supplement: Supplementary file 1 [file Table_1.DOCX]

**Fig.S1**

(A)

(B)

**Fig.S1 The triple-deletion mutant genetically stabile.**

The genomes of the extracted sublines were prepared as the template for PCR amplification with outer primers (SW-F/R) and inside primers (SN-F/R). Wildtype NTSN was set as the control to verify the deletion of the *actA*, *plcB* and *orfX* genes in NTSN∆*actA/plcB/orfX* F0, F5, F15 and F30. (A): Identification of NTSN and NTSN∆*actA/plcB/orfX* with SW-F/R and there is DNA Marker DL5000 in M lane; Lane 1 represents the PCR product of NTSN∆*actA/plcB/orfX* F0; Lane 2 presents the PCR product of wildtype NTSN; Lane 3 represents the PCR product of NTSN∆*actA/plcB/orfX* F5; Lane 4 represents the PCR product of NTSN∆*actA/plcB/orfX* F15; Lane 5 represents the PCR product of NTSN∆*actA/plcB/orfX* F30; (B): Identification of NTSN and NTSN∆*actA/plcB/orfX* with SN-F/R and M lane indicate the DNA Marker DL5000; Lane 1 represents the PCR product of NTSN∆*actA/plcB/orfX* wildtype NTSN; Lane 2 presents the PCR product of wildtype NTSN∆*actA/plcB/orfX* F0; Lane 3 represents the PCR product of NTSN∆*actA/plcB/orfX* F5; Lane 4 represents the PCR product of NTSN∆*actA/plcB/orfX* F15; Lane 5 represents the PCR product of NTSN∆*actA/plcB/orfX* F30.

**Table S1** Biochemical identification results of passage strains and NTSN wild-type strains

| **Biochemical tests and well** | **Wild-type**  **NTSN** | **Original generation（F0）** | **5^th^ generation（F5）** | **15^th^ generation（F15）** | **30^th^ generation（F30）** |
| --- | --- | --- | --- | --- | --- |
| **2 AMY** | **+** | **+** | **+** | **+** | **+** |
| **4 PIPLC** | **+** | **+** | **+** | **+** | **+** |
| **5 dXYL** | **-** | **-** | **-** | **-** | **-** |
| **8 ADH1** | **-** | **-** | **-** | **-** | **-** |
| **9 BGAL** | **-** | **-** | **-** | **-** | **-** |
| **11 AGLU** | **+** | **+** | **+** | **+** | **+** |
| **13 APPA** | **-** | **-** | **-** | **-** | **-** |
| **14 CDEX** | **+** | **+** | **+** | **+** | **+** |
| **15 AspA** | **-** | **-** | **-** | **-** | **-** |
| **16 BGAR** | **-** | **-** | **-** | **-** | **-** |
| **17 AMAN** | **+** | **+** | **+** | **+** | **+** |
| **19 PHOS** | **-** | **-** | **-** | **-** | **-** |
| **20 LeuA** | **+** | **+** | **+** | **+** | **+** |
| **23 ProA** | **-** | **-** | **-** | **-** | **-** |
| **24 BGURr** | **-** | **-** | **-** | **-** | **-** |
| **25 AGAL** | **-** | **-** | **-** | **-** | **-** |
| **26 PyrA** | **-** | **-** | **-** | **-** | **-** |
| **27 BGUR** | **-** | **-** | **-** | **-** | **-** |
| **28 AlaA** | **-** | **-** | **-** | **-** | **-** |
| **29 TyrA** | **+** | **+** | **+** | **+** | **+** |
| **30 dSOR** | **-** | **-** | **-** | **-** | **-** |
| **31 URE** | **-** | **-** | **-** | **-** | **-** |
| **32 POLYB** | **+** | **+** | **+** | **+** | **+** |
| **37 dGAL** | **-** | **-** | **-** | **-** | **-** |
| **38 dRIB** | **-** | **-** | **-** | **-** | **-** |
| **39 ILATk** | **-** | **-** | **-** | **-** | **-** |
| **42 LAC** | **+** | **+** | **+** | **+** | **+** |
| **44 NAG** | **+** | **+** | **+** | **+** | **+** |
| **45 dMAL** | **+** | **+** | **+** | **+** | **+** |
| **46 BACI** | **+** | **+** | **+** | **+** | **+** |
| **47 NOVO** | **+** | **+** | **+** | **+** | **+** |
| **50 NC6.5** | **+** | **+** | **+** | **+** | **+** |
| **52 dMAN** | **-** | **-** | **-** | **-** | **-** |
| **53 dMNE** | **+** | **+** | **+** | **+** | **+** |
| **54 MBdG** | **+** | **+** | **+** | **+** | **+** |
| **56 PUL** | **-** | **-** | **-** | **-** | **-** |
| **57 dRAF** | **-** | **-** | **-** | **-** | **-** |
| **58 O129R** | **+** | **+** | **+** | **+** | **+** |
| **59 SAL** | **+** | **+** | **+** | **+** | **+** |
| **60 SAC** | **-** | **+** | **+** | **+** | **+** |
| **62 dTRE** | **+** | **+** | **+** | **+** | **+** |
| **63 ADH2s** | **-** | **-** | **-** | **-** | **-** |
| **64 OPTO** | **+** | **+** | **+** | **+** | **+** |

These 43 physiological and biochemical characteristics of primary vaccine candidates NTSN∆*actA/plcB/orfX* (F0), NTSN∆*actA*/*plcB*/*orfX* 5th (F5),15th (F15), 30th (F30) and NTSN wild-type strains were examined using a microbial fully automated identification instrument. Primary NTSN∆*actA*/*plcB*/*orfX* vaccine candidates were consistent with 43 physiological and biochemical characteristics completely consistent of subculture strains.
